# The formidable task of fighting COVID-19 in Sudan

**DOI:** 10.11604/pamj.supp.2020.35.137.24984

**Published:** 2020-08-07

**Authors:** Yasir Ahmed Mohammed Elhadi, Yusuff Adebayo Adebisi, Khlood Fathi Hassan, Salma Elmukashfi Eltaher Mohammed, Xu Lin, Don Eliseo Lucero-Prisno III

**Affiliations:** 1 High Institute of Public Health, Department of Health Administration and Behavioral Sciences, Alexandria University, Alexandria, Egypt,; 2 Faculty of Pharmacy, University of Ibadan, Ibadan, Nigeria,; 3 Neonatal Intensive Care Unit, Saad Abu Alela Hospital, Khartoum, Sudan,; 4 Department of Public Health and Caring Sciences, Uppsala University, Uppsala, Sweden,; 5 Department of Thoracic Surgery, the First Affiliated Hospital, Zhejiang University School of Medicine, Hangzhou, P.R. China,; 6 Department of Global Health and Development, London School of Hygiene and Tropical Medicine, London, United Kingdom

**Keywords:** COVID-19, responses, challenges, Sudan, Africa, Public Health

## Abstract

Sudan is facing a formidable task of fighting COVID-19. The country is suddenly challenged by this health issue that will test its path towards peace, stability, and development. The fragile task of handling COVID-19 epidemic in Sudan is brought about by several factors such as the weak healthcare system and political conflicts, that have been intertwined with the recent regime. Even before the COVID-19 pandemic, there was already high unemployment, soaring inflation and lack of social protection and safety nets for its populace. The government has been trying its best to address the pandemic, however, much still needs to be done. Neglecting Sudan by the international community in terms of support towards containment of COVID-19 has grievous implications for transition out of military dictatorship and efforts to curb the pandemic globally. As no country is safe if all is not safe. It is essential that Sudan should leverage on innovations, country-compatible measures, and other tailor-made strategies for effective responses.

## Commentary

Sudan reported its first case of COVID-19 in Khartoum on 13 March 2020. It was a man who died the day before the case was confirmed. He visited the United Arab Emirates the week before. As of 12 July 2020, Sudan had 10,250 confirmed cases with 650 deaths [1]. The weak health system affected by years of neglect, lack of government investments doubly compounded by international sanctions, and waning interest from international donors make it difficult to face the gargantuan task of addressing COVID-19 in the country [2]. Sudan, with an estimated population of 43,849,260 [3] is suddenly faced with a relapse to political instability and potential conflict if this looming epidemic is not effectively addressed. Sudan is challenged by a health issue that will test its path towards peace, stability, and development. The fragile task of handling COVID-19 epidemic in Sudan is brought about by several factors that have been intertwined with the recent regime. Sudan is a chief example of country facing many dimensions of fragility with limited financial capacity to support its populace, lower state capacity to design and implement context-appropriate mitigation measures. The former ruling regime did not invest much on health institutions and on its healthcare workers as it prioritized other areas such as the military. There are 5.6 physicians per 10,000 population who are more concentrated in urban areas with 47.6 nurses and midwives serving 10,000 population [4]. There is only 1.2 hospitals and 13.5 primary health care centers per 100,000 residents in Sudan [5]. There is also a dire shortage of ICU beds in the country. Doctors take time to search for available beds just to accommodate critically ill patients who need advance medical apparatus. Personal protective equipment (PPE) are not easily accessible; and many hospital workers have been quarantined due to their contact with patients due to inadequate PPE. Late case reporting and high levels of stigma are also making COVID-19 containment challenging in Sudan.

Testing capacity has also been a challenge as there is a dearth of test kits. Aggravating the situation is medicine shortage in the country which is due to the inability of the government to buy them from abroad due to the limited dollar reserves of the country [6]. The weak economy due to the mismanagement of the past did not prepare Sudan for rough times. Even before the COVID-19 pandemic, there was already high unemployment, soaring inflation and lack of social protection and safety nets for its population. This has also led to serious brain drain of many professionals in the country. For instance, many Sudanese health professionals have migrated abroad to many countries around the world including Saudi Arabia and other countries in the Middle East. This presents a major challenge to Sudan´s healthcare system [7]. With a large proportion of Sudan´s population living below the poverty line, COVID-19 is likely to push more people into extreme poverty. This is likely to uptrend the percentage of the poor population in the country. This is worrisome in an era that the country attempted transition towards a civilian-led, democratic government. Many developing countries have been granted loan, however, Sudan is still struggling to access emergency funding [8]. Difficulties in accessing aid may be partly due to sanctions imposed by the United States as it considered Sudan a state sponsor of terrorism.

Neglecting Sudan in terms of support towards its containment of COVID-19 has grievous implications for transition out of military dictatorship and efforts to curb the pandemic worldwide, as no country is safe, if all is not safe. Furthermore, with poverty and hunger rife in Sudan, the possibility of getting hungrier is aggravated by the lockdowns and quarantines imposed by the government on the population thus closing their ability to earn daily wages and income from trade. The political situation is one major factor that increased the demand on the country´s health system. As the conflicts in some areas of the country has protracted, particularly in Darfur, South Kordofan and Blue Nile, these displaced nearly two million people in Sudan. Their situation in camps and settlements does not allow for good health conditions. This is aggravated by the 1.1 million refugees and migrants the country is already hosting. Once COVID-19 strikes among these populations, the demand will be too much to handle by the already stressed and stretched health system. The government has been trying its best to address the health issue. It tried to mitigate the problem by implementing new policies. For instance, the country stopped issuing visas for, and flights to, a number of countries at the early part of the pandemic and as the pandemic progressed to avoid further spread. It also closed its borders with Egypt. The government is trying to handle the situation by increasing the number of hospitals to accommodate the escalating number of patients in the capital Khartoum, however, the situation is not very well under control. Sudanese Prime Minister, Abdalla Hamdok, wrote to the United Nations acknowledging that COVID-19 is a major challenge to the country and requested for financial and technical support to tackle the pandemic [2].

Sudan has been suffering drought, war, political and economic instabilities for decades; and reform is urgently required in almost all sectors, particularly in health, economy, and education. The current government inherited from the previous ruling regime a decrepit health system and economic meltdown. As the current pandemic had threatened the strongest health systems around the world, this scenario represents a real threat and biggest challenge for authorities in Sudan to control the pandemic. The virus is starting to spread to all Sudanese states, where the situation is catastrophic and worse than the capital Khartoum. Health workers including doctors, nurses and laboratory technicians are unable to reach hospitals since there are no accommodations with in or near the hospitals. Frontline health workers are also testing positive COVID-19 while some private hospitals are shutting down. Some doctors have reported being beaten up and arrested by police officers enforcing the lockdown [6]. Limited PPE, unavailable means of transportation, and low wages are making the situation more difficult for healthcare providers to do their jobs thus further making containment challenging.

In order to limit the spread of the virus, authorities imposed the health emergency law and announced lockdown in the capital Khartoum. Unfortunately, many of Sudanese do not believe that the virus exists and that it had spread in Sudan. This further poses additional threat in complying with national health authorities stated guidelines and precautionary measures such as maintaining physical distancing, proper hand washing, and respiratory hygiene among others. Negative health beliefs, poor health literacy and misconceptions among Sudanese people, as a result of low level of education in the country, add to the problem. Under-estimation of the disease consequences, false and misleading information about the disease on social media are a major threat to containment efforts. The economic meltdown as well as locust swarm invasion in the country have led to food insecurity over the years [5]. Amid COVID-19 pandemic, people have to gather in rows to obtain food, which represents an excellent climate for the spread of the virus. The political conflict contributed much in the massive increase of cases. Different groups supporting political parties are spreading false news that the COVID-19 lockdown is part of a government conspiracy. This cajoles them to go out to demonstrate against the government. All these foretell the coming of the disaster and the country needs to increase its efforts for effective containment.

**Recommendations:** as it has been suggested that African countries need unique approaches in its efforts to curb the pandemic [9], Sudan needs to leverage on innovations, country-compatible measures, and other tailor-made strategies for effective responses. To support national efforts to limit the spread of coronavirus, Sudan´s national health authorities should continue to make efforts to ensure the necessary and critical information are available to the Sudanese public while ensuring adherence to precautionary measures such as the principles of physical distancing and avoiding large gatherings. Sudan´s national health authorities and other stakeholders should continue to share guidelines and recommendations with health authorities at the federal and state levels. It is also necessary to ramp up health awareness campaigns to improve COVID-19 health literacy in the country. Measures should also be put in place to ensure that rural communities in Sudan are not left behind in COVID-19 responses. Diagnostic testing is a challenge in many African countries [10], however, the testing capacity in Sudan is way below expectation, it is important that government should start to invest directly to increase diagnostic capacity as well as leveraging public-private partnerships to improve testing capacity. We also urge the international community to provide support to ensure effective containment of the pandemic in Sudan. As no country is safe if all is not safe.

## Conclusion

COVID-19 is causing several unprecedented crises in Sudan and is weighing heavily on a government that is already stressed with many development challenges. The country has many obstacles to hurdle in preparing the health system to effectively contain COVID-19 pandemic. It is now imperative that the government leads in this fight and continues to advance its efforts in response to COVID-19.
